# Strategies to Mitigate Establishment under the *Wolbachia* Incompatible Insect Technique

**DOI:** 10.3390/v14061132

**Published:** 2022-05-24

**Authors:** Stacy Soh, Soon Hoe Ho, Janet Ong, Annabel Seah, Borame Sue Dickens, Ken Wei Tan, Joel Ruihan Koo, Alex R. Cook, Shuzhen Sim, Cheong Huat Tan, Lee Ching Ng, Jue Tao Lim

**Affiliations:** 1 Environmental Health Institute, National Environment Agency, Singapore 138667, Singapore; stacy_soh@nea.gov.sg (S.S.); ho_soon_hoe@nea.gov.sg (S.H.H.); janet_ong@nea.gov.sg (J.O.); annabel_seah@nea.gov.sg (A.S.); sim_shuzhen@nea.gov.sg (S.S.); tan_cheong_huat@nea.gov.sg (C.H.T.); ng_lee_ching@nea.gov.sg (L.C.N.); 2 Saw Swee Hock School of Public Health, National University of Singapore, National University Health System, Singapore 117549, Singapore; ephdbsl@nus.edu.sg (B.S.D.); kenwei@nus.edu.sg (K.W.T.); ephkoor@nus.edu.sg (J.R.K.); ephcar@nus.edu.sg (A.R.C.); 3 School of Biological Sciences, Nanyang Technological University, Singapore 637551, Singapore

**Keywords:** *Wolbachia*, establishment, *Aedes aegypti*, compartmental modelling, simulation, Incompatible Insect Technique (IIT), Sterile Insect Technique (SIT), dengue, introgression

## Abstract

The Incompatible Insect Technique (IIT) strategy involves the release of male mosquitoes infected with the bacterium *Wolbachia*. Regular releases of male *Wolbachia*-infected mosquitoes can lead to the suppression of mosquito populations, thereby reducing the risk of transmission of vector-borne diseases such as dengue. However, due to imperfect sex-sorting under IIT, fertile *Wolbachia*-infected female mosquitoes may potentially be unintentionally released into the environment, which may result in replacement and failure to suppress the mosquito populations. As such, mitigating *Wolbachia* establishment requires a combination of IIT with other strategies. We introduced a simple compartmental model to simulate ex-ante mosquito population dynamics subjected to a *Wolbachia*-IIT programme. In silico, we explored the risk of replacement, and strategies that could mitigate the establishment of the released *Wolbachia* strain in the mosquito population. Our results suggest that mitigation may be achieved through the application of a sterile insect technique. Our simulations indicate that these interventions do not override the intended wild type suppression of the IIT approach. These findings will inform policy makers of possible ways to mitigate the potential establishment of *Wolbachia* using the IIT population control strategy.

## 1. Introduction

The *Aedes aegypti* (*Ae. aegypti*) mosquito is an efficient vector of several arboviruses, such as dengue, Zika, chikungunya and yellow fever [1]. Vector-borne diseases are a significant cause of morbidity and mortality, accounting for over 17% of the global burden of infectious diseases [2]. Urbanisation, along with population growth, sterilization, and climate change, has led to an intensification of these viruses in already endemic areas, an expanded geographical coverage of the *Ae. aegypti* mosquito, and an increased case burden in the tropics [3,4,5]. Dengue is a major arboviral disease that imposes a substantial burden across the globe, with an annual estimated infections of 390 million [6] globally, with an associated economic cost of around USD 8.9 billion [7]. Other important arboviruses, such as Zika and Yellow fever, which are also transmitted by the *Ae. aegypti* mosquito, have been spreading rapidly through the tropical and subtropical regions [8,9]. At present, considerable resources are being invested in vector control measures, such as source reduction and larviciding to suppress immature and adult mosquito numbers, which remain key strategies in mitigating the impact of arboviral disease transmission [10]. Their efficacy in suppressing mosquito populations, however, is limited, and persistent dengue outbreaks continue to occur [11,12,13,14]. This demonstrates the need for novel efficacious and cost-effective alternatives for vector control [15,16].

One such technology is the Incompatible Insect Technique (IIT) strategy [17], which involves the release of male *Ae. aegypti* mosquitoes infected with the bacterium *Wolbachia* [18]. The eggs produced by wild type *Ae. aegypti* female mosquitoes that have mated with *Wolbachia* carrying males are inviable due to cytoplasmic incompatibility [18], leading to the suppression of the mosquito population with regular release of the *Wolbachia*-infected *Ae. aegypti* mosquitoes over time. Suppression-based programmes have been implemented in the US [19], China [20] and Singapore [21,22] to complement existing vector control efforts. Crucially, the efficacy of the suppression approach relies on the over-flooding ratio of the male *Wolbachia-Aedes* mosquitoes to wild type females and the success of mating [23]. However, due to imperfect sex-sorting with various insect sex-sorting technologies, fertile *Wolbachia*-infected female mosquitoes may be unintentionally released into the environment [24]. The release of female mosquitoes may have the unintended effect of population replacement rather than elimination [25]. In combination, these factors reduce the desired outcome of elimination and pose open questions on how planners can mitigate *Wolbachia* establishment using the suppression approach.

To prevent the establishment of the *Wolbachia* strain in the field population, IIT has been combined with Sterile Insect Techniques (SIT), such that *Wolbachia*-infected mosquitoes undergo low-dose irradiation to sterilize residual females prior to release [26]. In general, females are more sensitive to irradiation compared to male mosquitoes [27,28], such that the irradiation dose required to achieve complete female sterility has minimal impact on male fitness [29,30]. Another approach to counter the unintended establishment includes release of a second bi-directionally cytoplasmic incompatible *Wolbachia* strain, which also exhibits cytoplasmic incompatibility with the wild type mosquitoes as well as with the established *Wolbachia* strain [31]. While these measures are biologically sound and have been demonstrated to be valid in laboratory settings, specific implementation measures, such as the duration and intensity in a competitive ecological setting, is challenging to establish. To this end, our study seeks to simulate possible strategies to mitigate potential establishment of *Wolbachia* in the wild type *Ae. aegypti* population through simulation.

Mathematical models have been used to simulate insect population dynamics over time to guide the timing and release numbers of *Wolbachia*-infected insects into the field population for SIT and IIT control strategies [32,33]. Knipling’s models of discrete-time dynamics and a simple geometric population growth model provided the original mathematical frameworks that have been used to successfully guide insect control programmes [23,34]. More sophisticated models involve differential equations to model mosquito abundance and genotypes [35]. Other attempts comprise spatial-temporal models with multiple life stages [36] and complex agent-based simulations [37], which have been developed to study the insect elimination process. To simulate changes in the mosquito populations over time, as well as possible release/intervention strategies for *Wolbachia*, we generalize an ecological model that incorporates mosquito transition dynamics in the aquatic and adult stages, mortality in the various stages, and multiple *Wolbachia* strains within a competitive ecological environment.

This model seeks to address key questions related to potential intervention strategies to counter the establishment of the *Wolbachia* strain under the suppression approach, namely:

1. How does the Female Release Error Rate (FRER) under sex sorting impact the likelihood of *Wolbachia* establishment under the suppression approach?
2. Do the proposed intervention strategies undermine the original suppression approach (i.e., how do they affect the wild type population)?
3. Are the proposed intervention strategies effective in countering the establishment of *Wolbachia*?
4. Are there other potential issues surrounding the proposed intervention strategies?

These simulations address important questions and provide insights into the efficacy of release strategies to counter the establishment of the *Wolbachia* in the wild type *Ae. aegypti* population in the environment. These findings may be used to support the adoption of an appropriate release strategy. As these simulations are exploratory in nature, the model structure and parameters may be adjusted according to estimates from scientific literature and field studies, and generalised to different settings.

## 2. Materials and Methods

To examine the efficacy of strategies to counteract the establishment of *Wolbachia* using a suppression approach, we developed a data driven framework to simulate the possible ecological dynamics of mosquito populations under several strategies. As the ecological trajectories differed under each strategy, we assessed these strategies separately and determined their efficacy by tracking the female mosquito population over time. Below, in Table 1, we define key terminologies used to describe the methods and results of the study.

### 2.1. Simulation of Wolbachia-IIT Programmes

Our study focused on a simulation of a *Wolbachia*-IIT programme, through the repeated release of male *Ae. Aegypti*, infected with *w*AlbB or *w*Mel *Wolbachia* into an environment comprising wild type mosquito populations. Both Wolbachia strains, *w*AlbB and *w*Mel have successfully been established in natural *Ae. aegypti* populations [39].

We first simulated a baseline scenario (S1) with no interventions in place, followed by a *Wolbachia-*IIT programme that employed a constant release strategy to suppress the wild type mosquito population using *w*AlbB male mosquitoes (S2). For this release strategy, we considered overflooding ratios of 10,000 and 60,000 (See Table 2). These overflooding ratios scale the number of *Wolbachia* infected mosquitoes released with respect to the initial uninfected adult wild type population size. The overflooding ratios were combined with three levels of separation fidelity determined by Female Release Error Rates (FRER; Table 2) of 10^−3^, 10^−9^ and 0, which correspond to the demonstrated rates achievable using next-generation mechanical sex separation as of 2021 [38,40] and with IIT-SIT respectively.

We considered two general strategies to counter the establishment of *Wolbachia*-infected female mosquitoes. First, we simulated the release of *Wolbachia*-infected male mosquitoes, with the unintentional females released rendered infertile via irradiation under the SIT-IIT (S3A). This served as a correction of the FRERs under the original suppression approach (S2), thereby reducing the chance of establishment where fertile females are unintentionally released in the environment due to imperfect sex-sorting. Second, we simulated the release of *Wolbachia*-infected male rendered infertile via irradiation under SIT-IIT (S3B), with the unintentional females released rendered infertile via irradiation as well. The sterilization by irradiation of both male and female *Wolbachia* mosquitoes reduces the likelihood viable offspring and fertile *Wolbachia* mosquitoes mating [40]. At present, the use of IIT-SIT S3A has already been trialed in Singapore and China [26,41]. Third, we simulated the release of male mosquitoes infected with a second *Wolbachia* strain (*w*Mel) to induce bi-directional cytoplasmic incompatibility with the first *Wolbachia* strain and wild type mosquito population (S4).

Additionally, we varied the release intensities, FRERs (Table 2) and intervention start and end dates (see Supplementary Material) to examine how quickly establishment may be mitigated and whether the former effect of wild-type elimination was moderated. In considering different intervention start and end dates, we explored two scenarios where (i) the interventions (S3A, S3B, S4) began after the end of the suppression approach (S2) and (ii) the interventions (S3A, S3B, S4) began before the end of the suppression approach (S2) (see Supplementary Material Table S2). In presenting our primary results in the following section, we refer to the scenario where the intervention strategies (S3A, S3B, S4) began after the end of the suppression approach unless stated otherwise.

We generated 1000 simulations for each scenario (S1–S4) to quantify the level of uncertainty in wild type suppression (S2) and intervention efficacy in countering establishment (S3A, S3B, S4). Each set of simulations was used to study the probabilities of: (i) unintended establishment of the first strain; (ii) wild type elimination, and (iii) eliminating establishment of the first strain. We incorporated parameter uncertainty for each simulation under every scenario by allowing each parameter to take a random draw from a uniform probability distribution over a plausible range. This allows for a plausible degree of variation that may be exhibited in a natural population. The resulting population trajectories produced under the simulations were therefore robust to a range of parameterisations and population outcomes.

### 2.2. Simulating Mosquito Populations over Time

We simulated changes in the mosquito populations over time using a discrete-time compartmental model, incorporating adult females (*F*), adult males (*M*) and an aggregated aquatic (*A*) stage that includes the egg, larvae and pupae stages in a competitive, well-mixed ecological environment. In this model, we incorporated three mosquito strains: the wild type and two different types of *Wolbachia* infected mosquitoes. The two *Wolbachia* considered were the *w*AlbB and *w*Mel strains in this study.

**Figure 1:**
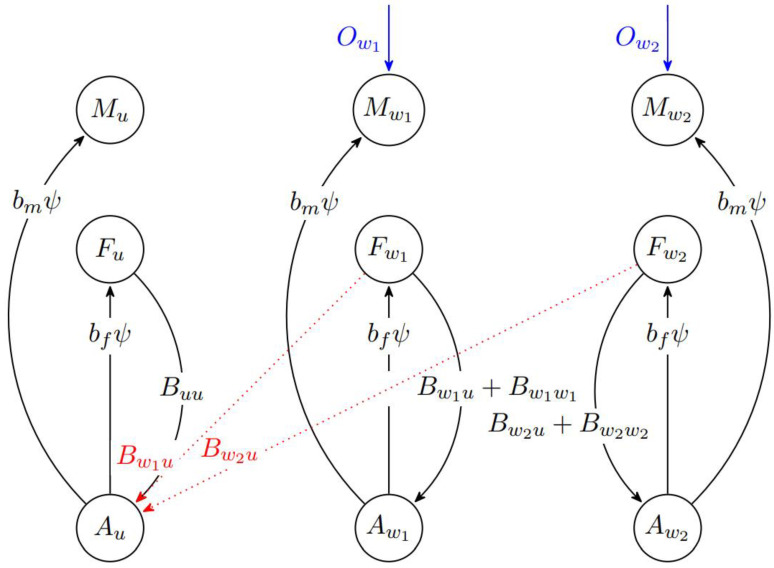
Schematic of the mosquito ecological model incorporating suppression and interventions to mitigate establishment. The circles represent specific mosquito populations, and the lines represent the transitions from one mosquito population to another, with the values within the lines representing transition rates between populations. The blue lines represent the release of male *Wolbachia* inoculated mosquitoes at rates $O_{w_{1}}$ or $O_{w_{2}}$. The birthing rates capture the uninfected offspring that are produced when the uninfected males mate with the uninfected females. When the *w*AlbB or *w*Mel-infected males mate with the uninfected females, inviable offspring are produced due to CI. Uninfected males mating with the infected *w*AlbB or *w*Mel-infected female produces a fraction of infected offspring by vertical transmission. Mating between *w*AlbB or *w*Mel-infected males with *w*AlbB or *w*Mel-infected females respectively produces a fraction of infected offspring. Mating between *w*AlbB-infected males (or *w*Mel-infected males) with *w*Mel females (or *w*AlbB females) resulted in inviable offspring due to CI.

The mosquito populations were grouped into nine compartments, starting with a susceptible, uninfected aquatic stage $A_{u}$ and infected aquatic stages $A_{w_{1}}$*,* $A_{w_{2}}$*,* where the subscripts $w_{1}$ and $w_{2}$ refer to the *w*AlbB and *w*Mel infected aquatic stage mosquitoes, respectively. Correspondingly, we denote $F_{u}$*,* $M_{u}$ as the uninfected female and male adult mosquitoes and $F_{w_{1}}$*,* $F_{w_{2}},\;M_{w_{1}}$*,* $M_{w_{2}}$ as the infected adult female and male adult mosquitoes (Figure 1). The eclosion rates of uninfected, *w*AlbB and *w*Mel mosquitoes are $\psi A_{u}$*,* $\psi A_{w_{1}},\;\psi A_{w_{2}},\;$ respectively. Death rates are given by ${µ}_{a}A_{u},\;{µ}_{a}A_{w_{1}},\;{µ}_{a}A_{w_{2}}$ for uninfected, *w*AlbB and *w*Mel mosquitoes in the aquatic stage respectively.

The parameters $b_{f}$ and $b_{m}$ govern the proportion of female and male adult mosquitoes being enclosed after the aquatic stage, respectively. After the transition to the adult stage (i.e., $F_{u}$, $F_{w_{1}}$*,* $F_{w_{2}}$*,* $M_{u}$*,* $M_{w_{1}}$*,* $M_{w_{2}}$), there is a probability of mating between adults of different sexes, subject to their respective populations at that point of time. Births are subject to constraints in the carrying capacity of the population. We consider the following equation ascertaining the effective number of aquatic stage births $B_{w_{1}u}$ between *w*AlbB infected female and uninfected wild-type male mosquitoes:

(1) $$B_{w_{1}u}=\left(1-c_{w_{1}u}\right)\;\;\varphi_{w}\;F_{w_{1}}m_{u}\;\xi \;\;\;\;$$

$$\mathrm{Where}\;\xi \;=\;1\;-\;\frac{N_{A}}{K_{A}}$$

where $c_{w_{1}u}$ denotes the degree of cytoplasmic incompatibility, and $m_{u}$ the proportion of uninfected wild type mosquitoes at that time point. Mating between irradiated male and female *Wolbachia* mosquitoes resulted in inviable offspring due to sterility. We assume 100% sterility following irradiation, consistent with previous studies that achieved full sterility under lab and semi-field conditions [41,42]. $N_{A}$ and $K_{A}$ denote the total number of aquatic mosquitoes in the population and the total aquatic carrying capacity of the population, respectively. Therefore, *ξ* governs the breeding constraints in the environment. This results in future aquatic stage mosquitoes being produced at a higher rate when the current population of aquatic mosquitoes is low and at a lower rate when the current population of aquatic mosquitoes is high. These constraints also provide a simple representation of higher aquatic stage mortality at higher aquatic populations. The multiple between the two adult stage mosquito populations $F_{w_{1}}m_{u}$ help ascertain the number of births $B_{w_{1}u}$ in the next time step. Each adult population has a respective birth equation subject to these constraints; their specifications are described in full detail in the Supplementary Material, together with all parameters used for the model obtained from literature-derived sources.

## 3. Results

To address the questions proposed by this study, we focused on a subset of the mosquito populations that are of key entomological and epidemiological interest, that is, the wild type, *w*AlbB and *w*Mel female mosquito populations. In Table 3 we show the following key findings regarding elimination of key mosquito populations:

### 3.1. How Does the Error Rate under Sex Sorting Impact the Likelihood of Wolbachia Establishment under the Suppression Approach?

**Figure 2:**
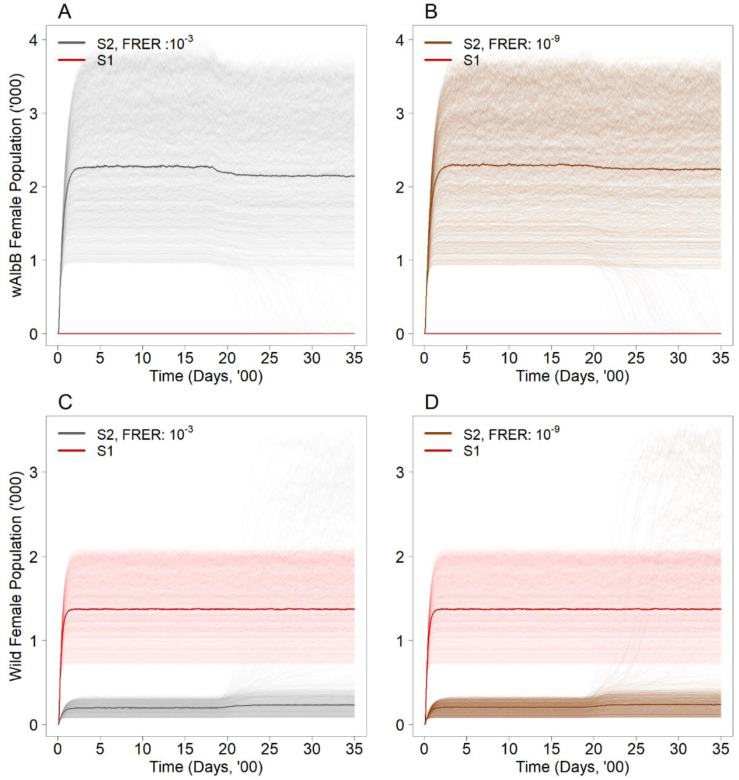
Number of *Wolbachia*-infected female mosquitoes (*w*AlbB) in the population with (**A**) FRER: 10^−3^ and (**B**) FRER: 10^−9^ under the suppression approach (S2) with the same male release intensity. Number of wild type female mosquitos in the population with (**C**) FRER: 10^−3^ and (**D**) FRER: 10^−9^ under the suppression approach (S2) with the same male release intensity. The lighter shade solid lines represent the 1000 simulations, and the dark solid lines represent the median of the 1000 simulations. The red solid line represents the baseline scenario (S1).

In general, the suppression approach (S2) with constant releases for 100 weeks resulted in the establishment of *w*AlbB even under very low female release error rates (FRERs). Under FRERs of 10^−3^ and 10^−9^, which correspond to the sex sorting error rates of current and next-generation sex sorting technologies [40,41], we found that establishment would occur under the simulated release of 10,000 male *Wolbachia* mosquitoes per week in an environment where the aquatic carrying capacity is 10,000 (Figure 2A,B). However, the established *w*AlbB population remained relatively constant between levels of 2210 (95% UI: 2152–2268) and 2141 (95% UI: 2083–2199) under both FRERs, demonstrating that establishment eventually stabilized at a certain level even under the continued constant release of *Wolbachia* male mosquitoes under different FRERs.

### 3.2. Do the Proposed Intervention Strategies Undermine the Original Suppression Approach (i.e., How Do They Affect the Wild Type Population)?

The suppression approach (S2) using the first strain at FRERs of 10^−3^ and 10^−9^ resulted in an 82.4% (95% UI: 80.0–85.0%) and 82.5% (95% UI: 79.7–85.4%) decline in the wild-type female population, respectively (Figure 2C,D), relative to the baseline (S1) equilibrium wild-type population. These results confirm the levels of suppression achieved on the wild type female mosquito population from ecological experiments [43,44].

**Figure 3:**
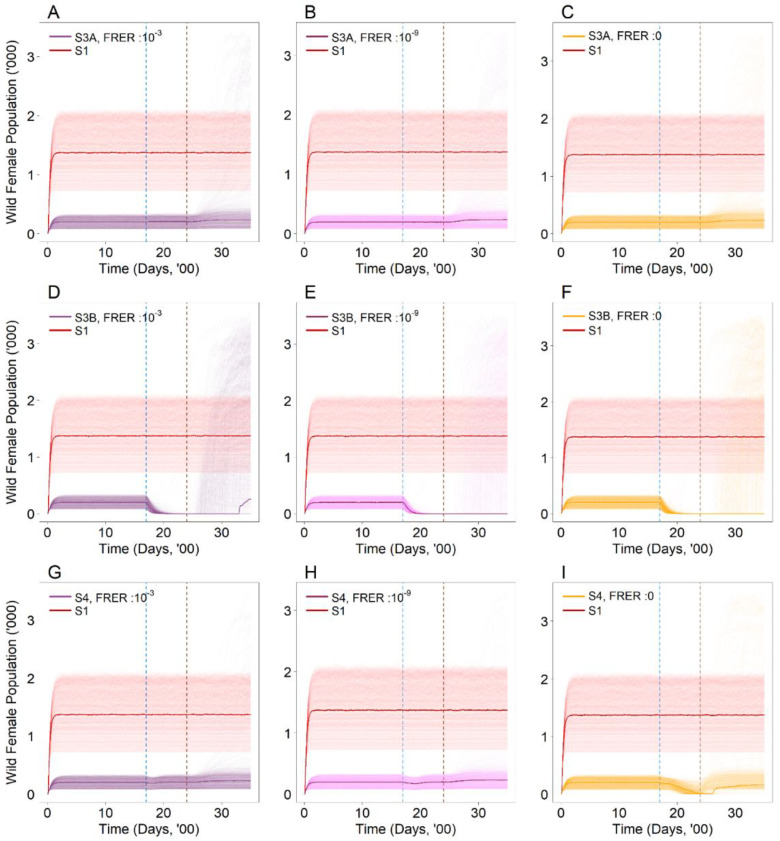
Number of wild type female mosquitoes in the population with (**A**) FRER: 10^−3^, (**B**) FRER: 10^−9^ and (**C**) FRER: 0 under the first strain female irradiation (S3A) with the same male release intensity. Number of wild type female mosquitoes in the population with (**D**) FRER: 10^−3^, (**E**) FRER: 10^−9^ and (**F**) FRER: 0 under the first strain male and female irradiation (S3B) with the same male release intensity. Number of wild type female mosquitoes in the population with (**G**) FRER: 10^−3^, (**H**) FRER: 10^−9^ and (**I**) FRER: 0 under the second strain introduction (S4) with the same male release intensity. The lighter shade solid lines represent the 1000 simulations, and the dark solid lines represent the median of the 1000 simulations. The blue and brown dotted vertical line represents the start and end of the intervention (S3A, S3B and S4) respectively. The red solid line represents the baseline scenario (S1).

Under SIT-IIT of female mosquitoes (S3A) with an FRER of 10^−9^ (Figure 3B), the equilibrium wild type female population remained suppressed, with a decline of 82.7% (95% UI: 81.0–84.3%) in the wild type female mosquito populations compared to the baseline of having no releases in place (S1) (Figure 3B). The equilibrium levels observed were similar to that of the pure suppression approach (S2) (Figure 2C,D). In addition, the decline and the eventual equilibrium levels in the wild type female population did not vary considerably under different FRERs for this particular strategy (S3A) (Figure 3A,C).

Under SIT-IIT of male and female mosquitoes (S3B) with varying FRERs, the wild type female population was eliminated at the simulation endpoint (Figure 3D–F). However, under an alternative intervention start and end point where the interventions began before the end of the suppression approach (see Supplementary Material), the wild type female population was only eliminated at smaller FRERs of 10^−9^ and 0, but not under an FRER of 10^−3^ (see Supplementary Material). This was due to a rebound in the population sometime after the intervention had ceased. In contrast, elimination was not observed under the pure suppression approach (S2) (Figure 2C,D).

Lastly, by introducing a second *Wolbachia* strain (S4) with non-zero FRERs, the equilibrium wild type female population remained suppressed, with a decline of between 82.9–83.2% in the wild type female mosquito populations compared to the baseline of having no releases in place (S1) (Figure 3G,H). These results were consistent under the alternative intervention start and end dates (see Supplementary Material). In contrast, under FRER = 0, the wild type female population was eliminated at the simulation endpoint (Figure 3I). However, under the alternative intervention start and end dates (see Supplementary Material), the wild type female population declined to low levels before stabilizing at non-zero levels at the simulation endpoint (see Supplementary Material). The equilibrium levels observed under non-zero FRERs (Figure 3G,H) were similar to that of the suppression approach (S2) (Figure 2C,D), while a zero-equilibrium endpoint was reached under FRER = 0 (Figure 3I).

Collectively, these results demonstrate that the intervention strategies do not undermine the suppression achieved under the original suppression strategy (S2), with the equilibrium endpoint levels for the wild type population stabilising at comparable or lower levels than observed before.

### 3.3. Are the Proposed Intervention Strategies Effective in Countering the Establishment of Wolbachia?

**Figure 4:**
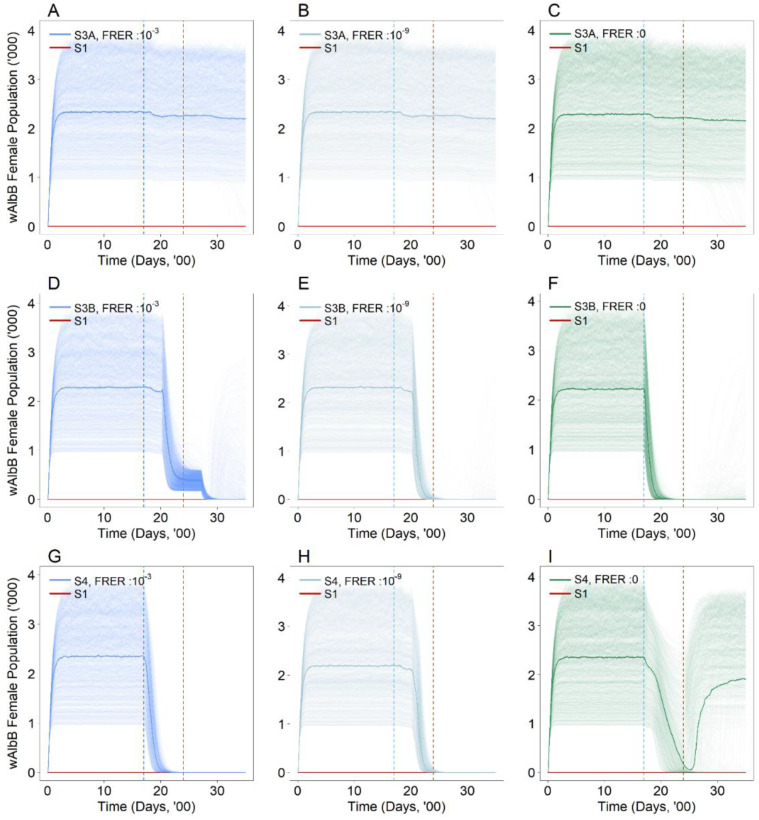
Number of *Wolbachia* (*w*AlbB) female mosquitoes in the population with (**A**) FRER: 10^−3^, (**B**) FRER: 10^−9^ and (**C**) FRER: 0 under the first strain female irradiation (S3A) with the same male release intensity. Number of wild type female mosquitoes in the population with (**D**) FRER: 10^−3^, (**E**) FRER: 10^−9^ and (**F**) FRER: 0 under the first strain male and female irradiation (S3B) with the same male release intensity. Number of wild type female mosquitoes in the population with (**G**) FRER: 10^−3^, (**H**) FRER: 10^−9^ and (**I**) FRER: 0 under the second strain introduction (S4) with the same male release intensity. The lighter shade solid lines represent the 1000 simulations, and the dark solid lines represent the median of the 1000 simulations. The blue and brown dotted vertical line represents the start and end of the intervention (S3A, S3B and S4) respectively. The red solid line represents the baseline scenario (S1).

Rendering the first strain *Wolbachia* female mosquitoes infertile by irradiation (S3A) did not mitigate the establishment of the original female *Wolbachia* strain under all FRERs (Figure 4A–C). These results were consistent under alternative intervention start and end dates (see Supplementary Material). Under S3A, only the females were rendered infertile. Mating between the fertile first strain *Wolbachia* males and the established *Wolbachia* females within the environment may have still occurred, preventing the elimination of these established *Wolbachia* females. However, under S3B where both males and females were sterilised by irradiation, offspring were not produced between the *Wolbachia-*infected males and females, resulting in the eventual elimination of the established female *Wolbachia* mosquitoes (Figure 4D–F). These results were consistent under alternative intervention start and end dates (see Supplementary Material). Likewise, the introduction of the second *Wolbachia* strain S4 resulted in the elimination of the established female *Wolbachia* mosquitoes (Figure 4G–I). Under alternative intervention start and end dates where the interventions began before the end of the suppression approach and at an FRER level of 0, the *Wolbachia* female population level dipped to near zero before re-establishing again at the simulation endpoint (see Supplementary Material). Again, this was due to a rebound in the population sometime after the intervention had ceased.

Under the use of irradiated first strain males and females S3B, the rate of elimination of the female *Wolbachia* population increased with a lower FRER (Figure 4D–F). This increased rate of elimination resulted in a reduction in the time (no. of days) required to bring down the female population and to eventually become eliminated. However, under the second strain introduction S4, the rate of elimination declined with a lower FRER (Figure 4G–I). This decline in the elimination rate resulted in a longer period of time (no. of days) required to bring down the female population and to eventually become eliminated. These findings were largely consistent under the alternative intervention start and end date scenario, apart from the second strain introduction S4 with an FRER of 0 where re-establishment occurred.

Under S3B, the released *Wolbachia* males were rendered sterile, which conferred sterility when mating with the wild type and the established *Wolbachia* females. Under S4, the introduction of males infected with the second *Wolbachia* strain resulted in bi-directional cytoplasmic-incompatibility with both the wild type and established *Wolbachia* females. These separate effects under the individual strategies worked to eliminate the established first strain *Wolbachia* female mosquitoes.

### 3.4. Are There Other Potential Issues Surrounding the Proposed Intervention Strategies?

**Figure 5:**
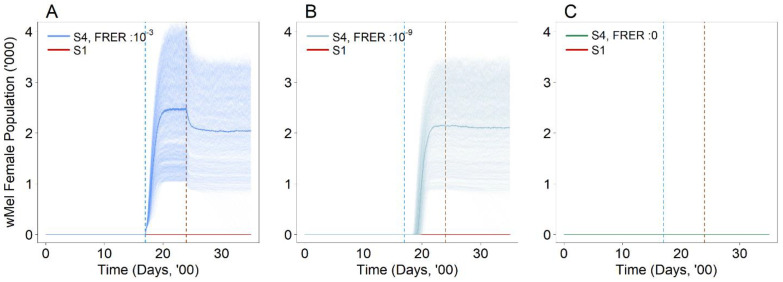
Number of second strain *Wolbachia* (*w*Mel) female mosquitoes in the population with (**A**) FRER: 10^−3^, (**B**) FRER: 10^−9^ and (**C**) FRER: 0 under the second strain introduction (S4) with the same male release intensity. The lighter shade solid lines represent the 1000 simulations, and the dark solid lines represent the median of the 1000 simulations. The blue and brown dotted vertical line represents the start and end of the intervention (S4) respectively. The red solid line represents the baseline scenario (S1).

While introducing the second *Wolbachia* strain (S4) was effective as an intervention to eliminate the established first strain *Wolbachia* female mosquitoes, the second *Wolbachia* strain might establish in the population if the FRER is non-zero (Figure 5A,B). This arises due to the unintended release of female second strain *Wolbachia* mosquitoes into the environment under imperfect sex separation and resulted in establishment even under a very low FRER of 10^−9^ (Figure 5B). Under non-zero FRERs, the eventual second strain *Wolbachia* female population mirrored that of the original establishment levels of the first *Wolbachia* strain (Figure 2A,B). Establishment of the second strain did not occur under zero FRER (Figure 5C).

**Figure 6:**
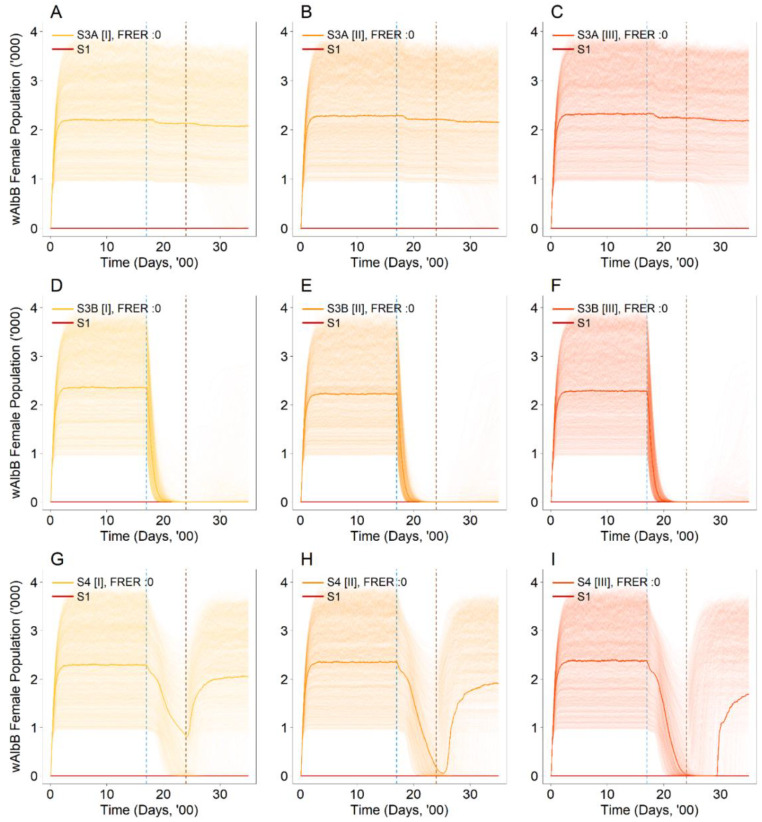
Number of *Wolbachia* (*w*AlbB) female mosquitoes in the population with FRER: 0 with (**A**) Reduced intensity of 40,000 releases per week (S3A [I]), (**B**) Baseline intensity of 60,000 releases per week (S3A [II]) and (**C**) Increased intensity of 80,000 releases per week (S3A [II]) of male releases under the first strain female irradiation (S3A). Number of *Wolbachia* (*w*AlbB) female mosquitoes in the population with FRER: 0 with (**D**) Reduced intensity of 40,000 releases per week (S3B [I]), (**E**) Baseline intensity of 60,000 releases per week (S3B [II]) and (**F**) Increased intensity of 80,000 releases per week (S3B [II]) of male releases under the first strain male and female irradiation (S3B). Number of *Wolbachia* (*w*AlbB) female mosquitoes in the population with FRER: 0 with (**G**) Reduced intensity of 40,000 releases per week (S3B [I]), (**H**) Baseline intensity of 60,000 releases per week (S3B [II]) and (**I**) Increased intensity of 80,000 releases per week (S3B [II]) of male releases under the second strain introduction (S4). The lighter shade solid lines represent the 1000 simulations, and the dark solid lines represent the median of the 1000 simulations. The blue and brown dotted vertical line represents the start and end of the intervention (S3A, S3B and S4) respectively. The red solid line represents the baseline scenario (S1).

In introducing the second *Wolbachia* strain (S4), adequate release numbers were required for the elimination of the original *Wolbachia* female population. Given fewer release numbers (S4 [I]) (Figure 6G), the *w*AlbB female mosquito population (i.e., original *Wolbachia* strain used for suppression) declined steadily during the intervention but did not reach elimination at the end of the intervention. Subsequently, the *w*AlbB female mosquito population rose again and stabilized at a non-zero levels at the simulation endpoint. At higher release numbers (S4 [II] and S4 [III]), (Figure 6H,I), the *w*AlbB female mosquito population was eliminated at a faster rate before the end of the intervention and stabilized at zero thereafter. These results demonstrate the importance of determining the necessary threshold for the male release numbers to ensure the complete and sustained elimination of the original *Wolbachia* strain.

## 4. Discussion

The *Wolbachia*-IIT strategy has been shown to be a promising method for suppressing mosquito populations [41,45] and our results validate these findings. This study, together with IIT field trials invites comparison with the SIT approach, which has also demonstrated success in suppressing mosquito populations [46]. However, under SIT, there are several drawbacks when the technique is employed independently [46]. Firstly, SIT comprises the release of female mosquitoes, which contributes to pest biting and potential pathogen transmission. Secondly, a constant balance between sterility and mating competitiveness is required, which may not allow for ideal mosquito suppression. Finally, irradiation alone only suppressed mosquito populations, but does not achieve wild-type elimination. A combination of SIT and IIT can circumvent these issues, which may allow for the desired effect of population elimination.

At baseline, we demonstrated a significant reduction in the wild type mosquito population (Figure 2C,D) of up to 83%, which is similar to field studies available in the current literature [26,40,41]. High levels of suppression achieved in the field can be attributed to the complementary effects of *Wolbachia*-IIT with conventional vector control measures [47], with high community acceptance in places where it is trialed [45,46,48,49,50] contributing in part to its success. Successfully suppressing the wild type population may increase the likelihood of establishment of the *Wolbachia* strain due to lowered competition from wild type mosquitoes in the environment. This could result in *Wolbachia* mosquitoes replacing the wild type population, jeopardizing the suppression effort and rendering the strategy unsuccessful. Intervention measures are therefore required to prevent the establishment of *Wolbachia* in wild type mosquito populations. As such, we sought to implement possible strategies for mitigating the establishment of the formerly released *Wolbachia* strain (*w*AlbB) within an appropriate modelling framework.

We presented several key strategies which may be used to mitigate the potential establishment of the released *Wolbachia* strain. Namely, (i) the persistent use of irradiated male and female Wolbachia mosquitoes via SIT-IIT (S3B) and (ii) the release of a second *Wolbachia* strain (*w*Mel, S4) after establishment of the former *Wolbachia w*AlbB has occurred. Under S3B, even if imperfect sex sorting (FRER *>* 0) persists together with SIT-IIT, the established *w*AlbB female mosquito population can be successfully eliminated. Similarly, under S4, the established female *w*AlbB female mosquito population can be successfully eliminated, but under non-zero FRERs for the *w*Mel releases, the establishment of *w*Mel female mosquito populations will occur. In both cases, the wild type mosquito population remains suppressed compared to the baseline levels, demonstrating that these interventions do not undermine the accrued suppression benefits from the originally released *Wolbachia* strain. These findings support the use of a hybrid approach [38], where areas with high dengue burden may benefit from the release of non-irradiated *Wolbachia* male mosquitoes to significantly reduce high mosquito populations, followed by the use of irradiated male and female mosquitoes (S3B) to counter the potential issue of establishment.

Among the various release ratios and female release error rates explored under S3B and S4, we found that higher release ratios were more effective in driving down the female population of the released *Wolbachia* strain (See Supplementary Material). These interventions, however, required a specific threshold value for the elimination of the *Wolbachia* strain (See Supplementary Material). To take over the established *Wolbachia* mosquito population’s niche—considering, for example, the intrinsic rate of population growth—the release of a minimum number of sterile or bi-directionally cytoplasmic incompatible mosquitoes may be required. The release numbers and intensity govern the probability of mating between the released sterile/bi-directionally cytoplasmic incompatible male mosquitoes with the established *Wolbachia* female mosquitoes. Consequently, whether the niche is taken over depends on the release numbers and intensity. Under fewer number of mosquitoes being released this threshold might not be achieved and the probability of mating between the released *Wolbachia* males and established *Wolbachia* females may be insufficient to take over this niche, preventing the successful elimination of the established *Wolbachia-*infected *Ae. aegypti* strain. While actual mosquito populations are difficult to ascertain in the real world, our results demonstrate the need to couple proposed interventions with entomological surveillance to assess the efficacy of interventions targeted at mitigating establishment and for the appropriate timing of intervention scale ups and/or rollbacks.

In our study, we explored the possibility of establishment under a constant release strategy and several interventions to mitigate this. Pagendam et al. highlighted the possibility for adaptive and crude adaptive releases under IIT to mitigate establishment [39]. However, implementing these strategies appears difficult in practice as they require release numbers to be tailored to time-specific population size estimates. Conversely, the same number of *Wolbachia*-infected males were released at every release event under the constant release approach in S3B,4, negating the need for real-time and accurate population surveillance. Scaling up such a strategy in terms of release numbers and frequency would be far simpler, without additional resources required for operational purposes.

We followed Pagendam et al. [39], Jansen et al. [51] and Magori et al. [39] by incorporating stochasticity into our model simulations by nesting the biologically plausible range of parameters in each simulation run. This has several benefits over purely deterministic models, such as the incorporation of uncertainty intervals in the evolution of each mosquito population and, therefore, the likelihood under which establishment may be mitigated given a specific intervention. Crucially, stochastic models such as ours can help capture important facets in mosquito population dynamics, such as the uncertainty in each mosquito’s mortality and birth rates, thereby mimicking the large range of possible outcomes observed in the field; these can drastically change the eventual behaviour of the mosquito populations in the simulation, especially when particular subgroups of interest become minute in size.

In tackling the question about strategies to mitigate *Wolbachia* establishment, we required a simulation framework that could easily parameterize various facets about the mosquito populations. Existing stochastic models were difficult to adapt, with Jansen et al. [51] assuming constant population size over time—which does not hold under the IIT approach—and with Magori et al. [37] requiring that multiple spatially explicit parameters be identified, such as the breeding container locations and numbers. The three-strain compartmental model introduced in this study relied on a literature-derived range of parameters and could be used to draw realistic conclusions about the dynamics of general mosquito populations in an environment where IIT, SIT-IIT and/or a second *Wolbachia* strain strategies are employed.

Empirical evidence suggests that mosquito larval breeding sites are often fragmented [52], with the degree of population mixing determined by mosquito dispersal and oviposition behaviour [52]. In our study, we adopted a simple representation of density-dependent competition in a large well-mixed population from a single breeding site. We also assumed a linear increase in larval mortality and birth rates. These assumptions likely oversimplify the complex dynamics of the density-dependent competition across a fragmented landscape, which may have implications on the ecological trajectories of the mosquito population. Absolute population numbers were also taken to be arbitrary as any non-zero starting population in the compartmental model eventually converges to an equilibrium population based on model parameters. Therefore, the efficacy of the strategies in our study were evaluated based only after mosquito populations converged to equilibrium and was taken relative to each respective mosquito population, before and after the intervention occurred.

In our study, an independent IIT programme had been initiated to suppress mosquito populations, resulting in the issue of establishment of the Wolbachia strain in the field [38,41]. We therefore explored strategies such as SIT and the introduction of a second bi-directionally cytoplasmic incompatible Wolbachia strain to complement the existing IIT strategy and mitigate establishment. Future programmes should, however, take pre-emptive measures to prevent the issue of establishment, such as a highly efficient sex-sorting system [40] or by employing SIT-ITT from the beginning to ensure population elimination and prevent establishment.

## 5. Conclusions

To mitigate establishment of the released *Wolbachia* strain, our study suggests the irradiation of male and female *Wolbachia* mosquitoes under SIT-IIT with all FRERs can be effective. Our simulations indicate that this intervention does not override the intended effect of wild type suppression under the IIT approach.

## Figures and Tables

**Table 1:** Glossary of terminology.

Term	Definition
Incompatible Insect Technique (IIT)	A technique whereby *Wolbachia*-infected male mosquitoes are released to mate with wild type females, preventing the formation of viable offspring due to cytoplasmic incompatibility. See [20].
Sterile Insect Technique and Incompatible Insect Technique (SIT-IIT)	A technique whereby sterile *Wolbachia*-infected male mosquitoes are released to mate with wild type females, preventing viable offspring from forming. The sterility is due to irradiation [20]. This may include the unintentional release of sterile irradiated females under non-zero FRERs.
Constant Release Strategy	A strategy whereby an equal number of *Wolbachia-*infected mosquitoes is released at every release event throughout the IIT programme. The number of individuals released is not modified through monitoring of the wild type population. Number of *Wolbachia*-infected mosquitoes released per event = overflooding ratio × male population at start of programme. See [38].
Female Release Error Rate (FRER)	The rate at which fertile *Wolbachia*-infected females are accidentally released with *Wolbachia*-infected males into the field, due to errors in the separation of sexes during the production phase.
Eliminated	A mosquito population is considered to have been eliminated when there are no mosquitoes alive in either the aquatic or adult stages in the model at the end of the simulation. See [38].
Establishment	Having a stable female mosquito population infected with the released *Wolbachia* strain used for suppression at the simulation endpoint.

**Table 2:** Scenarios considered for modelling mosquito populations.

Scenario	Release Intensity ^1^/Overflooding Ratio ^2^	FRER ^3^
Units	’0,000	
Baseline (S1)	–	–
Suppression Approach (S2)	1	10^−3^, 10^−9^
SIT-IIT Female (S3A)	4, 6, 8	
SIT-IIT Male + Female (S3B)	4, 6, 8	10^−3^, 10^−9^, 0
Second Strain Introduction (S4)	4, 6, 8	10^−3^, 10^−9^, 0

^1^ Denotes the number of *Wolbachia* mosquitoes released per week. We focused on the simulations for S3A, S3B and. S4 under the release of 60,000 male mosquitoes per week unless specified otherwise. ^2^ Denotes the ratio of male *Wolbachia* mosquitoes released in relation to the initial wild type male population. ^3^ FRER denotes the female release error rates, i.e., the proportion of *Wolbachia* females released in comparison to the number of male *Wolbachia* mosquitoes released under the suppression strategy.

**Table 3:** Successful elimination of specific mosquito populations at simulation endpoint. “Yes” indicates the successful elimination of the respective mosquito population. “No” indicates that there was no elimination of the respective mosquito population.“N/A” indicates that the population was not released under that scenario.

	Population								
Strategy	S3A			S3B			S4		
Error Rates	10^−3^	10^−9^	0	10^−3^	10^−9^	0	10^−3^	10^−9^	0
*w*AlbB Female	No	No	No	Yes	Yes	Yes	Yes	Yes	Yes
Wild Female	No	No	No	Yes	Yes	Yes	No	No	Yes
*w*Mel Female	N/A	N/A	N/A	N/A	N/A	N/A	No	No	Yes

## Data Availability

All data and parameters used in this study are available from the Supplementary Material and https://github.com/stacysoh/wolbEstab (accessed on 7 April 2022).

## Supplementary Materials

The following supporting information can be downloaded at: https://www.mdpi.com/article/10.3390/v14061132/s1, Table S1: Parameters used for core ecological model; Table S2: Different intervention start/endpoints used for simulation; Figure S1: Alternative scenario wildtype female mosquito population; Figure S2: Alternative scenario *w*Alb female population; Figure S3: Alternative scenario *w*Mel female mosquito population. Reference [53] was cited in the Supplementary Material.
