# Supplementary material for: Strategies to Mitigate Establishment under the Wolbachia Incompatible Insect Technique
Source: Viruses. 2022 May 24;14(6):1132. doi: 10.3390/v14061132 (PMC9229438; doi:10.3390/v14061132)
Supplement: Supplementary file 1 [file viruses-14-01132-s001.zip › viruses-1696351-supplementary.pdf]

Supplementary Information to "Strategies to Mitigate Establishment Under the *Wolbachia* Incompatible Insect Technique"

Stacy Soh[1], Soon Hoe Ho[1], Janet Ong[1], Annabel Seah[1],
Borame Sue Dickens[2], Ken Wei Tan[2], Joel Ruihan Koo[2], Alex R Cook[2],
Shuzhen Sim[1], Cheong Huat Tan[1],Lee Ching Ng[1,5], Jue Tao Lim[1,2,*]

[1]Environmental Health Institute, National Environment Agency, Singapore, Singapore 138667, Singapore
[2]Saw Swee Hock School of Public Health, National University of Singapore, Singapore 117549, Singapore
[5]School of Biological Sciences, Nanyang Technological University, Singapore 637551, Singapore
[*]Corresponding author: juetao@nus.gov.sg
May 27, 2022

Contents

1 Ecological model incorporating wildtype and 2 *Wolbachia* strains **2**
1.1 Aquatic Stage Equations 2
1.2 Adult Stage Equations 3
1.3 Birth Equations 4
1.4 Parameters used for core ecological model 5

2 Different intervention start/endpoints used for simulation **6**

3 Alternative scenario wildtype female mosquito population **7**

4 Alternative scenario *w*Alb female population **8**

5 Alternative scenario *w*Mel female mosquito population **9**

1 Ecological model incorporating wildtype and 2 *Wolbachia* strains

We employ a compartmental model which may incorporate wildtype mosquitoes and 2 *Wolbachia* strains in the population. The following sections describe (1) joint aquatic stages for mosquitoes (2) adult stages for female and male mosquitoes (3) equations parameterizing births of new aquatic stage mosquitoes. We lastly detail the parameters used for our model.

1.1 Aquatic Stage Equations

This model incorporates a joint aquatic stage $A_{\odot}$ which comprises the egg, pupae and larvae stages, where the subscript $\odot$ represents either the wild type u, wAlbB w_1 or wMel w_2 strains in the population. In the susceptible, uninfected aquatic stage A_u, births occur at rate $B_{uu} + V_u\left(B_{w_1u} + B_{w_1w_1} + B_{w_2u} + B_{w_2w_2}\right)$ and deaths occur with rate $\mu_a A_u$. They transition to the uninfected adult stage with rate ψA_u. Similarly, in the infected aquatic stages A_{w_1}, A_{w_2}, births occur at rates $V_w\left(B_{w_1u} + B_{w_1w_1}\right)$ and $V_w\left(B_{w_2u} + B_{w_2w_2}\right)$ respectively. With transitions out of the compartment governed by deaths with parameter μ_a and eclosion with parameter ψ:

$$
\begin{aligned}
\frac{dA_u}{dt} &= B_{uu} + V_u\left(B_{w_1u} + B_{w_1w_1} + B_{w_2u} + B_{w_2w_2}\right) - \mu_a A_u - \psi A_u \\
\frac{dA_{w_1}}{dt} &= V_w\left(B_{w_1u} + B_{w_1w_1}\right) - \mu_a A_{w_1} - \psi A_{w_1} \\
\frac{dA_{w_2}}{dt} &= V_w\left(B_{w_2u} + B_{w_2w_2}\right) - \mu_a A_{w_2} - \psi A_{w_2}
\end{aligned}
\tag{S1}
$$

1.2 Adult Stage Equations

In the adult stage, $F_{\odot}, M_{\odot}$ refers respectively to female and male adult mosquitoes. Adult stage mosquitoes enter the compartmental with rates $b\psi A_{\odot}$, where b refers to the proportion of births which are either male (b_m) or female (b_f). Similarly, they exit the compartment with rates $\mu_{\odot}F_{\odot}, \mu_{\odot}M_{\odot}$. To model suppression or intervention strategies, the compartments $\frac{dM_{w1}}{dt}, \frac{dM_{w2}}{dt}$ contain the terms $O_{w_1,t}, O_{w_2,t}$ which denote the number of mosquitoes being released at the timepoint t. Note that we model release intensities and frequencies of various quantities for each simulation as described in the main text.

Crucially, we monitor the absolute value of female *Wolbachia* mosquitoes F_{w_1}, F_{w_2} at the end time of the simulation, to examine whether establishment has either occurred or been mitigated given a specific scenario.

$$
\begin{aligned}
\frac{dF_u}{dt} &= b_f\psi A_u - \mu_{f_u}F_u \\
\frac{dF_{w_1}}{dt} &= b_f\psi A_{w_1} - \mu_{f_{w_1}}F_{w_1} \\
\frac{dF_{w_2}}{dt} &= b_f\psi A_{w_2} - \mu_{f_{w_2}}F_{w_2} \\
\frac{dM_u}{dt} &= b_m\psi A_u - \mu_{m_u}M_u \\
\frac{dM_{w_1}}{dt} &= b_m\psi A_{w_1} - \mu_{m_{w_1}}M_{w_1} + O_{w_1,t} \\
\frac{dM_{w_2}}{dt} &= b_m\psi A_{w_2} - \mu_{m_{w_2}}M_{w_2} + O_{w_2,t}
\end{aligned}
\tag{S2}
$$

1.3 Birth Equations

Aquatic stage births, as mentioned in the preceding section, are crucially governed by B, the egg laying rate for each possible mating pair between males and females, wildtype and *Wolbachia* mosquitoes of either strain. Therefore, we denote $B_{\odot\otimes}$ as the egg laying rate for the female mosquito of type $\odot$ and male mosquito of type $\otimes$.

The birth rate depends on (1) the egg laying rate $\phi_{\odot}$, the number of female mosquitoes $F_{\odot}$ and the proportion of males $m_{\otimes}$ in the population at that point of time. Egg laying is also subject to a competitive ecological environment and this is parameterized by ξ, which denotes the remaining proportion that the environment can carry aquati stage mosquitoes in the environment. Lastly, for mating between wild type and *Wolbachia* mosquitoes, we parameterize cytoplasmic incompatibility (and therefore the suppressive effect *Wolbachia* has) by the term $c_{\odot\otimes}$:

$$
\begin{aligned}
B_{uu} &= \phi_u F_u m_u \xi \\
B_{uw_1} &= (1 - c_{uw_1})\phi_u F_u m_{w_1} \xi \\
B_{uw_2} &= (1 - c_{uw_2})\phi_u F_u m_{w_2} \xi \\
B_{w_1u} &= (1 - c_{w_1u})\phi_w F_{w_1} m_u \xi \\
B_{w_2u} &= (1 - c_{w_2u})\phi_w F_{w_2} m_u \xi \\
B_{w_1w_1} &= (1 - c_{w_1w_1})\phi_w F_{w_1} m_{w_1} \xi \\
B_{w_2w_2} &= (1 - c_{w_2w_2})\phi_w F_{w_2} m_{w_2} \xi \\
B_{w_1w_2} &= (1 - c_{w_1w_2})\phi_w \left(F_{w_1} m_{w_2} + F_{w_2} m_{w_1}\right) \xi = 0 \\
\text{where} \quad \xi &= 1 - \frac{N_A}{K_A}
\end{aligned}
\tag{S3}
$$

1.4 Parameters used for core ecological model

Parameter	Notation	Value	Source
Uninfected male, female, and aquatic mosquitoes	F_U, M_U, A_U	–	–
wAlbB male, female, and aquatic mosquitoes	$F_{w_1}, M_{w_1}, A_{w_1}$	–	–
wMel male, female, and aquatic mosquitoes	$F_{w_2}, M_{w_2}, A_{w_2}$	–	–
Per capita development rate of mosquito eggs	ψ	0.01	[53]
Death rate (aquatic stage)	μ_a	0.02	[53]
Female death rate (adult stage, wild type)	μ_{f_u}	0.027 – 0.070	[53]
Female death rate (adult stage, wAlbB)	$\mu_{f_{w_1}}$	0.023 – 0.062	[53]
Female death rate (adult stage, wMel)	$\mu_{f_{w_2}}$	0.023 – 0.152	[53]
Male death rate (adult stage, wild type)	μ_{m_u}	0.028 – 0.080	[53]
Male death rate (adult stage, wAlbB)	$\mu_{m_{w_1}}$	0.021 – 0.052	[53]
Male death rate (adult stage, wMel)	$\mu_{m_{w_2}}$	0.022 – 0.055	[53]
Fraction male births	b_m	0.5	–
Per capita egg laying rate, Wolb-free mosquito eggs	ϕ_u	76.188 – 81.522	[53]
Per capita egg laying rate, wAlbB-infected mosquito eggs	ϕ_{w_1}	83.893 – 88.043	[53]
Per capita egg laying rate, wMel-infected mosquito eggs	ϕ_{w_2}	93.972 – 99.308	[53]
Population carrying capacity	K_A	10000	–
Cytoplasmic incompatibility			
Uninfected female, wMel male	c_{uw_1}	1	[53]
Uninfected female, wAlb male	c_{uw_2}	1	[53]
wMel female, uninfected male	c_{w_1u}	0.337 – 0.417	[53]
wAlb female, uninfected male	c_{w_2u}	0.182 – 0.262	[53]
wMel female, wMel male	$c_{w_1w_1}$	0.165 – 0.285	[53]
wAlb female, wAlb male	$c_{w_2w_2}$	0.12 – 0.20	[53]
wAlb female, wMel male	$c_{w_1w_2}$	1	[53]
wMel female, wAlb male	$c_{w_2w_1}$	1	[53]

Table S1: Parameters used for core ecological model

2 Different intervention start/endpoints used for simulation

Scenario<br>Units	Start point<br>Time (t)	End point<br>Time (t)
Baseline (**S1**)	–	–
Suppression Approach (**S2**)	20	1,820
[1] SIT Female (**S3A**)	2,020	2,720
[1] SIT Male+Female (**S3B**)	2,020	2,720
[1] Second Strain Introduction (**S4**)	2,020	2,720
[2] SIT Female (**S3A**)	1,700	2,400
[2] SIT Male+Female (**S3B**)	1,700	2,400
[2] Second Strain Introduction (**S4**)	1,700	2,400

Table S2: The two ([1] and [2]) scenarios with different intervention start and end points in the simulations considered for modelling mosquito populations. The simulation time frame of t=0 to T=3,500 was considered.
[1] represents the main scenario with the respective start and end points for the strategies to mitigate establishment (**S3A, S3B** and **S4**). The interventions began after the end of the suppression approach (**S2**).
[2] represents an alternative intervention start and end point for the strategies to mitigate establishment (**S3A, S3B** and **S4**) where the interventions began before the end of the suppression approach (**S2**).

3 Alternative scenario wildtype female mosquito population

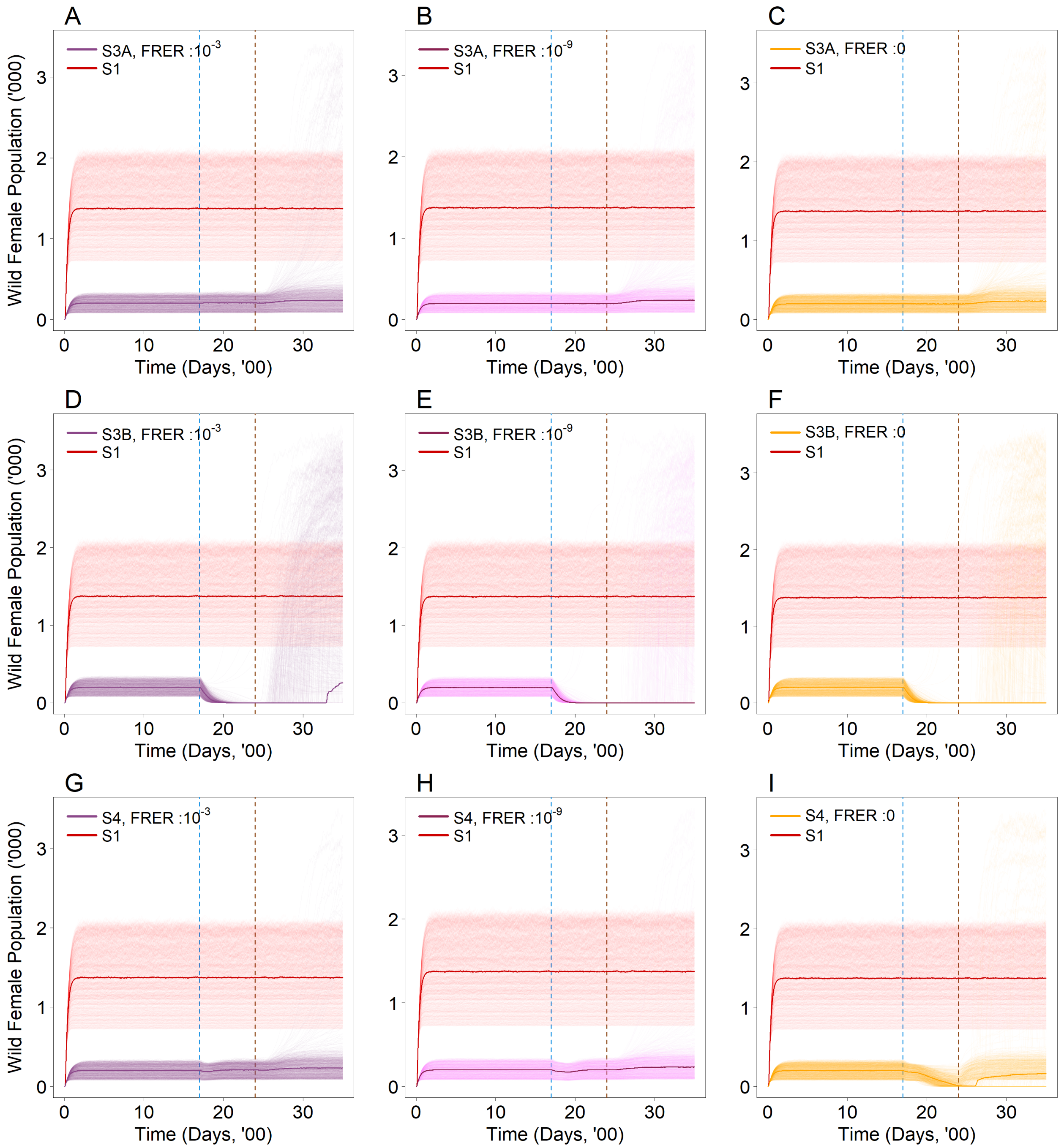

Figure S1: Number of wild type female mosquitoes in the population with (A) FRER: 10^{-3}, (B) FRER: 10^{-9} and (C) FRER: 0 under the first strain female irradiation (**S3A**) with the same male release intensity under the alternative start and end dates. Middle row from left to right: Number of wild type female mosquitoes in the population with (D) FRER: 10^{-3}, (E) FRER: 10^{-9} and (F) FRER: 0 under the first strain male and female irradiation (**S3B**) with the same male release intensity under the alternative start and end dates. Bottom row from left to right: Number of wild type female mosquitoes in the population with (G) FRER: 10^{-3}, (H) FRER: 10^{-9} and (I) FRER: 0 under the second strain introduction (**S4**) with the same male release intensity under the alternative start and end dates. The lighter shade solid lines represent the 1000 simulations and the dark solid lines represent the median of the 1000 simulations. The blue dotted vertical line represents the start of the intervention (**S3A, S3B** and **S4**). The red solid line represents the baseline scenario (**S1**).

4 Alternative scenario *w*Alb female population

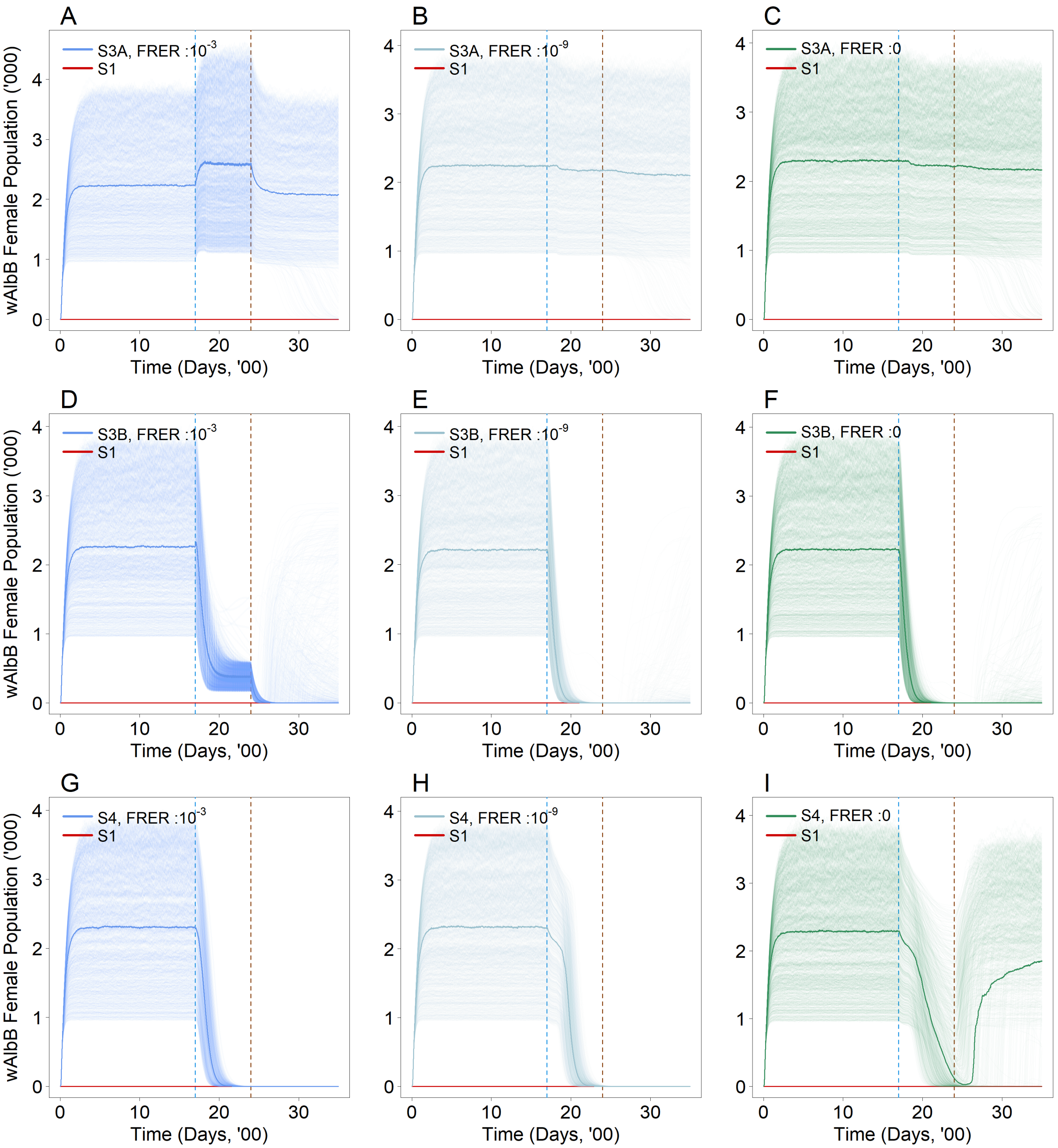

Figure S2: Number of *Wolbachia* (*wAlbB*) female mosquitoes in the population with (A) FRER: 10^{-3}, (B) FRER: 10^{-9} and (C) FRER: 0 under the first strain female irradiation (**S3A**) with the same male release intensity under the alternative start and end dates. Middle row from left to right: Number of wild type female mosquitoes in the population with (D) FRER: 10^{-3}, (E) FRER: 10^{-9} and (F) FRER: 0 under the first strain male and female irradiation (**S3B**) with the same male release intensity under the alternative start and end dates. Bottom row from left to right: Number of wild type female mosquitoes in the population with (G) FRER: 10^{-3}, (H) FRER: 10^{-9} and (I) FRER: 0 under the second strain introduction (**S4**) with the same male release intensity under the alternative start and end dates. The lighter shade solid lines represent the 1000 simulations and the dark solid lines represent the median of the 1000 simulations. The blue dotted vertical line represents the start of the intervention (**S3A, S3B** and **S4**). The red solid line represents the baseline scenario (**S1**).

5 Alternative scenario *w*Mel female mosquito population

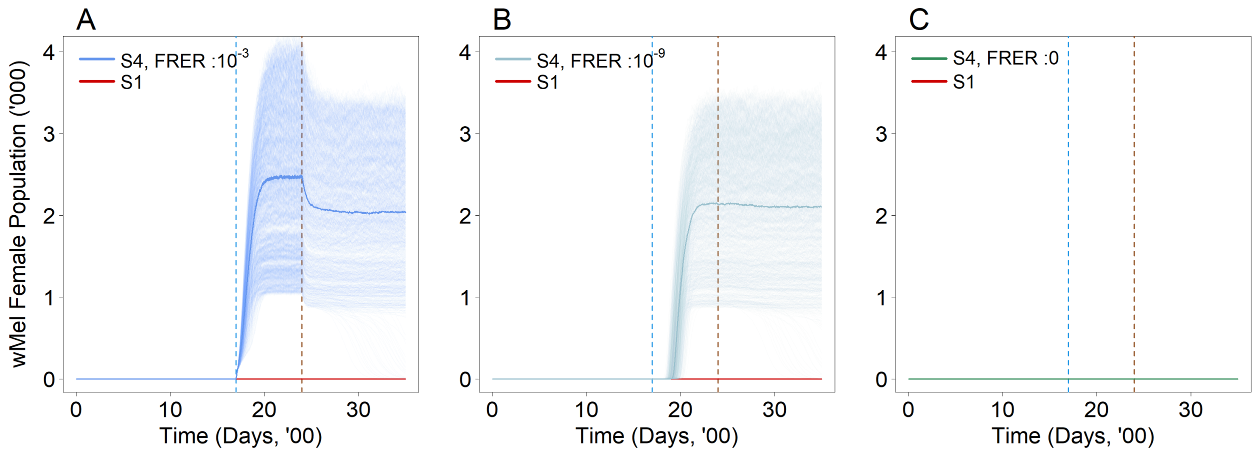

Figure S3: Number of second strain *Wolbachia* (*wMel*) female mosquitoes in the population with (A) FRER: 10^{-3}, (B) FRER: 10^{-9} and (C) FRER: 0 under the second strain introduction (**S4**) with the same male release intensity under the alternative start and end dates. The lighter shade solid lines represent the 1000 simulations and the dark solid lines represent the median of the 1000 simulations. The blue dotted vertical line represents the start of the intervention (**S4**). The red solid line represents the baseline scenario (**S1**).
